# Methyltransferase 3 Mediated miRNA m6A Methylation Promotes Stress Granule Formation in the Early Stage of Acute Ischemic Stroke

**DOI:** 10.3389/fnmol.2020.00103

**Published:** 2020-06-05

**Authors:** Wenwen Si, Yi Li, Shanyu Ye, Zhen Li, Yangping Liu, Weihong Kuang, Dongfeng Chen, Meiling Zhu

**Affiliations:** ^1^Shenzhen Bao’an Traditional Chinese Medicine Hospital (Group), Guangzhou University of Chinese Medicine, Shenzhen, China; ^2^The First Clinical Medical College, Guangzhou University of Chinese Medicine, Guangzhou, China; ^3^Department of Anatomy, The Research Center of Basic Integrative Medicine, Guangzhou University of Chinese Medicine, Guangzhou Higher Education Mega Center, Guangzhou, China; ^4^The Second Clinical Medical College, Guangdong Medical University, Dongguan, China; ^5^Shenzhen Hospital of Integrated Traditional Chinese and Western Medicine, Shenzhen, China

**Keywords:** METTL3, miR-335, Erf1, stress granules, acute ischemic stroke

## Abstract

The modification of methyltransferase-like (METTL) enzymes plays important roles in various cellular responses by regulating microRNA expression. However, how m6A modification is involved in stress granule (SG) formation in the early stage of acute ischemic stroke by affecting the biogenesis processing of microRNAs remains unclear. Here, we established a middle cerebral artery occlusion (MCAO) model in rats and an oxygen-glucose deprivation/reperfusion (OGD/R) model in primary cortical neurons and PC12 cells to explore the potential mechanism between m6A modification and SG formation. The *in vivo* results showed that the level of infarction and apoptosis increased while SG formation decreased significantly within the ischemic cortex with improved reperfusion time after 2 h of ischemia. Consistent with the *in vivo* data, an inverse association between the apoptosis level and SG formation was observed in PC12 cells during the reperfusion period after 6 h of OGD stimulation. Both *in vivo* and *in vitro* results showed that the expression of METTL3 protein, m6A and miR-335 was significantly decreased with the reperfusion period. Overexpression of the METTL3 and METTL3 gene-knockdown in PC12 cells were achieved via plasmid transfection and CRISPR-Cas9 technology, respectively. Overexpression or knockdown of METTL3 in oxygen-glucose deprivation of PC12 cells resulted in functional maturation of miR-335, SG formation and apoptosis levels. In addition, we found that miR-335 enhanced SG formation through degradation of the mRNA of the eukaryotic translation termination factor (Erf1). In conclusion, we found that METTL3-mediated m6A methylation increases the maturation of miR-335, which promotes SG formation and reduces the apoptosis level of injury neurons and cells, and provides a potential therapeutic strategy for AIS.

## Introduction

The acute ischemic stroke (AIS) is a severe neurological disease with high incidence, disability and mortality, which brings enormous psychological and economic burden to patients and the social economy (Manning et al., 2014; Fisher and Saver, 2015; Fraser, 2016). Intravenous recombinant tissue plasminogen activator (tPA) and endovascular thrombectomy (EVT) are the only effective treatments available for AIS (Lee et al., 2018). However, due to the narrow therapeutic window, patients with AIS may fail to gain the benefits of acute treatments (Nogueira et al., 2018). Cerebral ischemia/reperfusion (I/R) injury rapidly triggers different types of programmed cell death in neurons, such as apoptosis, autophagy and programmed necrosis, and this injury is inevitable and irreversible (Li et al., 2018). Therefore, it is vital to increase the resistance of neurons to ischemic conditions in the early stage of AIS when mildly injured neurons have not entered the process of apoptosis or necrosis (Ovesen et al., 2014).

Since the pathogenesis of AIS involves complex physiological and pathological processes, it is challenging to develop effective therapies and biological markers for the treatment in the early phase of the disease (Khandelwal et al., 2016; Zerna et al., 2016; Catanese et al., 2017). When neurons are exposed to ischemic and hypoxic microenvironments surrounding the ischemic brain tissue, the transient translation arrest occurs in the ischemic region, which reduces the misfolding of proteins and mistranslation of mRNA, thus inhibiting the necrosis of brain tissue and neuronal death (DeGracia and Hu, 2007; DeGracia et al., 2008; Vosler et al., 2011). Interestingly, the translation arrest of proteins is also an essential condition for the production of stress granules (SGs) (Beckham and Parker, 2008; Anderson and Kedersha, 2009). SGs are non-membrane aggregates of proteins and mRNAs, produced in the cytoplasm under various harmful environmental stimuli (Bhattacharyya et al., 2006; Buchan and Parker, 2009). SGs can stall immediately and transiently mRNA translation to protect valuable mRNAs and proteins from the injury of the harmful environment, thus improving the survival rate of cells in the early stage of AIS (Takahashi et al., 2013; Waris et al., 2014; Arimoto-Matsuzaki et al., 2016). However, the mechanism of SG formation in the early stage of AIS remains unclear.

Mature miRNAs are produced from primary microRNAs (pri-miRNAs) of several 100 base lengths through digestion with a series of nucleases (Murchison and Hannon, 2004; Winter et al., 2009). Recent evidence suggests that methyltransferase 3 (METTL3) increases the production of mature miRNA by adding a m6A modification to the primary miRNAs (pri-miRNAs), which makes it easier to be identified and digested by the DiGeorge syndrome critical region 8 (DGCR8) and Drosha enzyme (Alarcon et al., 2015; Han et al., 2019). In addition, it was found that METTL3 knockout decreased the binding activity between DGCR8 and pri-miRNA, leading to decreased expression of mature miRNAs (Alarcon et al., 2015). Our previous studies showed that miR-335 promotes the production of stress granules by targeting the Rho-associated coiled-coil forming protein kinase 2 (ROCK2), and its expression is reduced in AIS specificity (Si et al., 2019). Therefore, we hypothesized that m6A modification is involved in SG formation in the early stage of AIS by affecting the biogenesis processing of miR-335.

In this study, we used a rat MCAO (middle cerebral artery occlusion) model and an OGD/R model of PC12 cells and primary cortical neurons, to investigate METTL3-mediated m6A methylation of miR-335 in SG formation at the early stage of AIS.

## Materials and Methods

### Experimental Animals and MCAO Models

All animal experiments were approved by the Ethical Committee of Guangzhou University of Chinese Medicine. A total of 108 male Sprague-Dawley (*SD*: 230–260 g; 8–9-weeks old) rats were randomly divided into six groups (*n* = 18 rats/group): the sham group (sham operation); R-0 h group (MCAO for 2 h and reperfusion for 0 h); R-6 h group (MCAO for 2 h and reperfusion for 6 h); R-12 h group (MCAO for 2 h and reperfusion for 12 h); R-18 h group (MCAO for 2 h and reperfusion for 18 h); and R-24 h group (MCAO for 2 h and reperfusion for 24 h). The MCAO model was established as previously described (Si et al., 2019). Briefly, after a median incision on the neck, the left common carotid artery (CCA), internal carotid artery (ICA), and external carotid artery (ECA) were isolated. The left CCA and the ECA were ligated. Next, a silicone-coated suture was introduced into the left ICA through the ECA until it reached the middle cerebral artery to block the local blood flow for 2 h. After 2 h of MCAO, the suture was removed gently for blood reperfusion and the animals were placed on a heating pad to maintain body temperature at approximately ∼36.5°C until recovery from anesthesia. The animals were placed in a breeding room where humidity and temperature were under control and maintained in a 12-h light/dark cycle.

### 2, 3, 5-Triphenyl-Tetrazolium Chloride (TTC) Staining

Carbon dioxide asphyxiation was performed to sacrifice the animals. Whole brains were immediately removed and coronally cut into 2-mm thickness sections on ice. Brain slices were then stained in 2% TTC solution (Sigma-Aldrich, Merck KGaA, Darmstadt, Germany) for 20 min. The pale area of the hemisphere was defined as the infarct area and the infarct volume was calculated with Image J software (United States National Institutes of Health).

### Histological Analysis

After MCAO surgery, rats in each group were anesthetized through intraperitoneal injection by 100 mg/kg ketamine and 10 mg/kg xylazine. Through the injection of the cardiac aorta, blood washing and brain tissue fixation were performed using normal cold saline and 4% paraformaldehyde solution, respectively. Frozen (10 μm thickness) and paraffin section (3 μm thickness) of fixed brains were used for terminal deoxynucleotidyl transferase dUTP nick end labeling (TUNEL) staining and haematoxylin/eosin (HE) staining, respectively. TUNEL staining was performed using *In Situ* Cell Death Detection Kit (Sigma-Aldrich, Merck KGaA, Darmstadt, Germany) following the manufacturer’s the instructions. The apoptotic cells were identified through green fluorescence and the nuclei were counterstained with DAPI. Images were acquired using confocal laser scanning microscope (LSM 800, Zeiss, Germany). The percentage of positive cells was calculated with the Image-Pro Plus software (Version 6.0, United States). HE staining was conducted according to standard protocols and the histopathological observations were documented using a light microscope (Leica DMI400; Leica Microsystems GmbH, Wetzlar, Germany) at 400 X magnification and photographed.

### Extraction of Total RNA and RT-qPCR

Total RNA from the core area tissue of ischemic cerebral cortex or cell lysates was extracted and purified using the Direct-zol RNA Kit (ZYMO research, Irvine, CA, United States) according to the manufacturer’s protocol. Reverse transcription and qPCR assays were performed using the RT reagent kit (Takara Biotechnology, Co., Dalian, China) and the Light Cycler 480 SYBR Green I Master (Roche Diagnostics, GmbH, Mannheim, Germany), respectively. The Real-Time PCR Detection System (CFX96, Bio-Rad, United States) was used to detect the fluorescence of PCR products. Each experiment was replicated three times and data are presented as the mean ± SEM.

### Dot Blot Assay of Total RNA

Total RNA from the core area tissue of ischemic cerebral cortex or cell was denatured at 95°C to disrupt secondary structures. Denatured RNA (100 ng) was immediately chilled on ice and then crosslinked to the Hybond-N + membrane (GE Healthcare, Madison, WI, United States). The membrane was blocked with blocking buffer and then incubated with anti-m6A antibody (Abcam, Cambridge, United Kingdom; 1:500 dilution; 1 μg/mL) and goat anti-rabbit IgG-HRP (Abcam; 1:5000 dilution; 40 ng/mL). The ECL Substrate (Thermo Fisher Scientific, Waltham, MA, United States) was used to visualize the bands. Image acquisition was performed with the Tanon-6200 gel imaging system (Tanon, Shanghai, China). ImageJ software was used to measure the light intensity. Each experiment was replicated three times and data are presented as the mean ± SEM.

### Western Blotting Analysis

The cerebral cortex (ischemic core area) and cells were lysed with RIPA lysis buffer on ice for 20 min. After centrifugation at 10,000 × *g* for 20 min at 4°C, total proteins were harvested. The bicinchoninic acid assay (BCA) was used to determine the concentration of total proteins. The total protein (30 μg) was separated with 10% SDS-PAGE and transferred to a 0.45 um PVDF membrane (Millipore, Darmstadt, Germany). After blocking with 5% BSA, the membrane was incubated with primary and secondary antibodies. The ECL substrate (Pierce, Thermo Fisher Scientific, Inc., United States) was used to visualize the bands. Each experiment was replicated three times and data are presented as the mean ± SEM.

### Primary Cortical Neurons Culture

We cultured primary cortical neurons from neonatal rodent cortex (Sprague-Dawley rats, postnatal day 1) as previously described (Wang et al., 2014a, b). In brief, the P1 (postnatal day 1) neonatal rodent cortex was cut into a small piece (1 mm cube) and digested with trypsin at 0.25%. To obtain primary cortical neurons, the digested tissues were filtered through a 70 μm cell strainer (BD Biosciences, Bedford, MA, United States), and then cultivated for 24 h in fresh Dulbecco’s modified Eagle’s medium (DMEM, Gibco, Thermo Fisher Scientific, Inc., Waltham, MA, United States). After 24 h culture, neurons were incubated in neurobasal medium containing 1% B27 (Gibco, Thermo Fisher Scientific, Inc.) and 1% Pen Strep (Gibco, Thermo Fisher Scientific, Inc.). We renewed half of the medium every 2 days for 2 weeks. Finally, the neurons were subjected to OGD/R treatment. Each experiment was replicated three times and data are presented as the mean ± SEM.

### Oxygen-Glucose Deprivation/Reperfusion (OGD/R) Injury

Primary cortical neurons and PC12 cells (ATCC, Manassas, VA, United States) underwent OGD/R injury to construct the *in vitro* cell model of AIS. The OGD/R stimulation was performed as described previously. Briefly, neurons and PC12 cells were gently washed with PBS three times, incubated in glucose-free RPMI 1640 medium (Gibco; Thermo Fisher Scientific, Inc.) and then placed in an anaerobic chamber (Coy Laboratory Products, Inc., Grass Lake, MI, United States) to produce OGD stimulation under an atmosphere of 95% N_2_/5% CO_2_ at 37°C for 6 h. The OGD was terminated by replacing the glucose-free medium with normal medium and returning cells to normoxic conditions (5% CO_2_ and 95% air) for reperfusion at 37°C for 24 h. Primary cortical neurons and PC12 cells were incubated in neurobasal medium containing 1% B27 or RPMI 1640 medium with 5% fetal bovine serum under normoxic conditions for different reperfusion times (0, 6, 12, 18, and 24 h), respectively. Each experiment was replicated three times and data are presented as the mean ± SEM.

### Immunofluorescence Staining

The core components of SG, T-cell intracellular antigen-1 (TIA1), and GTPase-activating protein-binding protein 1 (G3BP1) are considered direct indicators of SG formation. Therefore, we used a double-labeled immunofluorescence (G3BP1 and TIA1) assay to observe SG generation in the cortex, PC12 cells, and primary cortical neurons. For immunofluorescent staining, frozen sections (10 μm thick) of the brain and cells (PC12 cells and primary cortical neurons) mounted on cover glass were fixed with 4% paraformaldehyde (PFA) for 20 min, permeabilized in 0.3% Triton X-100 solution for 10 min, blocked with blocking solution (5% BSA and 10% goat serum) for 1 h and finally incubated with primary antibodies (rabbit anti-TIA1 antibody, 1:1000, Abcam; mouse anti-G3BP1 antibody, 1:1000, Abcam, Cambridge, United Kingdom) and secondary antibodies (Alexa Fluor488-conjugated goat anti-rabbit antibody, 1:500, Abcam; Alexa Fluor555-conjugated goat anti-mouse antibody,1:500, Abcam) in the dark. DAPI staining for 10 min was used to label nuclei. Images were acquired using a confocal laser scanning microscope (LSM 800, Zeiss, Oberkochen, Germany). Each experiment was replicated three times and data are presented as the mean ± SEM.

### Flow Cytometry

The Apoptosis Detection kit (BD Biosciences, Bedford, MA, United States) was used to detect apoptosis in PC12 cells. In brief, PC12 cells (1 × 10^6^ cells) were digested with 0.25% trypsin (EDTA-free, Gibco, United States) and resuspended in 1 mL 1X binding buffer. Annexin V and propidium iodide were added to the cell suspension and incubated for 10 min in the dark. CytoFLEX flow cytometry (Beckman Coulter, Inc., United States) were performed to detect the fluorescence intensity of the cells. Each experiment was replicated three times and data are presented as the mean ± SEM.

### METTL3 and Erf1 Knockdown in PC12 Cells

Targeting sequences for CRISPR knockdown were designed using the CRISPR tool^1^. The knockdown of METTL3 in PC12 cells was generated with CRISPR-Cas9 gene editing system by targeting the following sequence: 5′-GCTTAGGGCCACTAGAGGTAGGG-3′. The target sequence for knockdown of Erf1 was 5′-GCT CATTAAGAGCTTGGAGGCGG-3′. Briefly, complementary oligonucleotides of METTL3 or Erf1 with *Bsa*I (New England Biolabs, Beijing, China) restriction sites for guide RNAs (gRNAs) were synthesized and inserted into the pGL3-U6-gRNA plasmid (a kind gift from Prof. Jiankui Zhou) and confirmed by sequencing. PC12 cells were seeded in 6-well plates (1 × 10^6^ cells/well). After 24 h cultivation, cells were transfected with 10 μg pST1374-Cas9-ZF-NLS (a kind gift from Prof. Jiankui Zhou) and 10 μg pGL3-U6-gRNA (either pGL3-U6-METTL3-gRNA or pGL3-U6-Erf1-gRNA) plasmid using 20 μl Lipofectamine 3000 and up to 250 μl Opti-MEM/well. After 48 h of transfection, RPMI-1640 complete medium with a concentration of 2 μg/mL of puromycin was added to the transfected cells to select positive clones. After selection of single-cell clones, positive clones were cultured to obtain the appropriate number of cells for further analysis. The primer sequences and plasmid maps are provided in Supplementary Material S2. Each experiment was replicated three times and data are presented as the mean ± SEM.

### Overexpression of METTL3 in PC12 Cells

The overexpression plasmids of METTL3 (pCDH-CMV-METTL3) used in this study were designed and constructed. According to the manufacturer’s instructions, PC12 cells (1 × 10^5^ cells) were transfected with 10 μg pCDH-CMV-METTL3 plasmids (Shenzhen Huaan Ping Kang Bio Technology, Inc., Shenzhen, China) with using Lipofectamine 3000 (Invitrogen; Thermo Fisher Scientific, Inc.). The empty pCDH-CMV plasmid was used as a negative control (NC). Each experiment was replicated three times and data are presented as the mean ± SEM.

### Dual-Luciferase Reporter System

Bioinformatics predictions (TargetScan and miRBase) were used to describe the targeting between miR-335 and the 3′-UTR of the eukaryotic translation termination factor 1 (Erf1). The psiCHECK2- Erf1 plasmid, which included miR-335 binding sites, was used to perform dual-luciferase reporter gene assay. PC12 cells (0.2 × 10^5^) were seeded on the cover glass, and then transfected with 100 ng psiCHECK2-Erf1 plasmids and 100 nmol/L miR-335 mimics using Lipofectamine 3000 (Invitrogen, United States). The cells were harvested 6 h after transfection and assayed using the dual-luciferase reporter gene assay kit (Promega, United States) according to the manufacturer’s instructions. Firefly luciferase was used for normalization. Each experiment was replicated three times and data are presented as the mean ± SEM.

### Transfection of the miRNA-335 Mimic and Inhibitor in PC12 Cells

MiR-335 mimic (micrON miRNA agomir; 5′-ucaagagc aauaacgaaaaaugu-3′) miR-335 mimic negative control (mimic NC) and miR-335 inhibitor (micrOFF miRNA antagomir; 5′-acauuuuucguuauugcucuuga-3′), miR-335 inhibitor negative control (inhibitor NC) were purchased from Guangzhou RiboBio, Co. (Guangzhou, China). PC12 cells (1 × 10^5^ cells) were transfected with miR-335 mimic (50 nM, final concentration), mimic NC (50 nM, final concentration), miR-335 inhibitor (100 nM, final concentration) and inhibitor NC (100 nM, final concentration) using Lipofectamine 3000 (Invitrogen; Thermo Fisher Scientific, Inc.). After 24 h of transfection, total protein and RNA were extracted from cells to perform western blotting and RT-PCR assays. Each experiment was replicated three times and data are presented as the mean ± SEM.

### RNA-Binding Protein Immunoprecipitation (RIP)

We performed RIP analysis using an EZ-Magna RIP kit (Millipore, Billerica, MA, United States) and specific antibodies (METTL3, Rabbit pAb, Proteintech, Wuhan, China; m6A, Rabbit mAb, Abcam) following the manufacturer’s instructions. The methods for reverse transcription and qPCR assays were identical to the RT-qPCR method described above. The Real-Time PCR Detection System (CFX96, Bio-Rad, United States) was used to detect the fluorescence value of PCR products. GAPDH mRNA was used as internal control for pri-miR-335, pre-miR-335, and U6 for mature miR-335. The amplification protocol was as follows: 95°C for 1 min; 35 cycles of 95°C for 30 s, 60°C for 30 s, 72°C for 30 s. Fold Enrichment = 2^(–△^^△^^*Ct[normalized* RIP])^;^△^^△^ Ct[normalized RIP] = ^△^ Ct[normalized RIP]-^△^ Ct[normalized IgG];^△^ Ct[normalized RIP] = (Average Ct[RIP]-(Average Ct[Input]-log_2_ [Input Dilution Factor])). Each experiment was replicated three times and data are presented as the mean ± SEM. Primers are shown as follows:

pri-miR-335 (398 bp) Forward: 5′-CCACACTCGGGAC ATTGAGT-3′;
pri-miR-335 (398 bp) Reverse: 5′-TGGCACCTATCTC CAAATGCT-3′.
pre-miR-335 (91 bp) Forward: 5′-GGCGGGGGTCAA GAGC-3′;
pre-miR-335 (91 bp) Reverse: 5′-TGGCTATAACAAATG AGAGGAGGTC-3′.
mature-miR-335 RT: 5′-GTCGTATCCAGTGCAGGGTC CGAGGTATTCGCACTGGATACGACACATTT-3′;
mature-miR-335 Forward: 5′-CGGCGCTCAAGAGCAA TAACGAA-3′;
mature-miR-335 Reverse: 5′-ATCCAGTGCAGGGTCCG AGG-3′.
U6 RT: 5′-GTCGTATCCAGTGCAGGGTCCGAGGTATT CGCACTGGATACGACAAAATA-3′;
U6 Forward: 5′-AGAGAAGATTAGCATGGCCCCTG-3′;
U6 Reverse: 5′-ATCCAGTGCAGGGTCCGAGG-3′.
GAPDH Forward: 5′-GGTGGACCTCATGGCCTACA-3′;
GAPDH Reverse: 5′-CTCTCTTGCTCTCAGTATCCTT GCT-3′.

### Statistical Analysis

The experimental data are presented as the mean ± SEM. SPSS 20.0 software (IBM, Corp., Somers, NY, United States) was used for statistical analyses. The normality of values was tested with the Shapiro–Wilk normality test. All figures were produced using GraphPad Prism (Version 6.0; GraphPad Software, Inc., La Jolla, CA, United States). A two-tailed unpaired *t*-test or non-parametric Mann–Whitney *U*-test was used to analyze the difference between the two groups. One-way ANOVA, two-way ANOVA, or Kruskal–Wallis non-parametric test were performed to analyze the difference between more than two groups followed by *post hoc* comparison (Tukey’s multiple comparisons test). A *P*-value of < 0.05 was considered statistically significant.

## Results

### The Inverse Relation Between SG Formation and Ischemic Injury in the Ischemic Cortex of MCAO Rats

**FIGURE 1:**
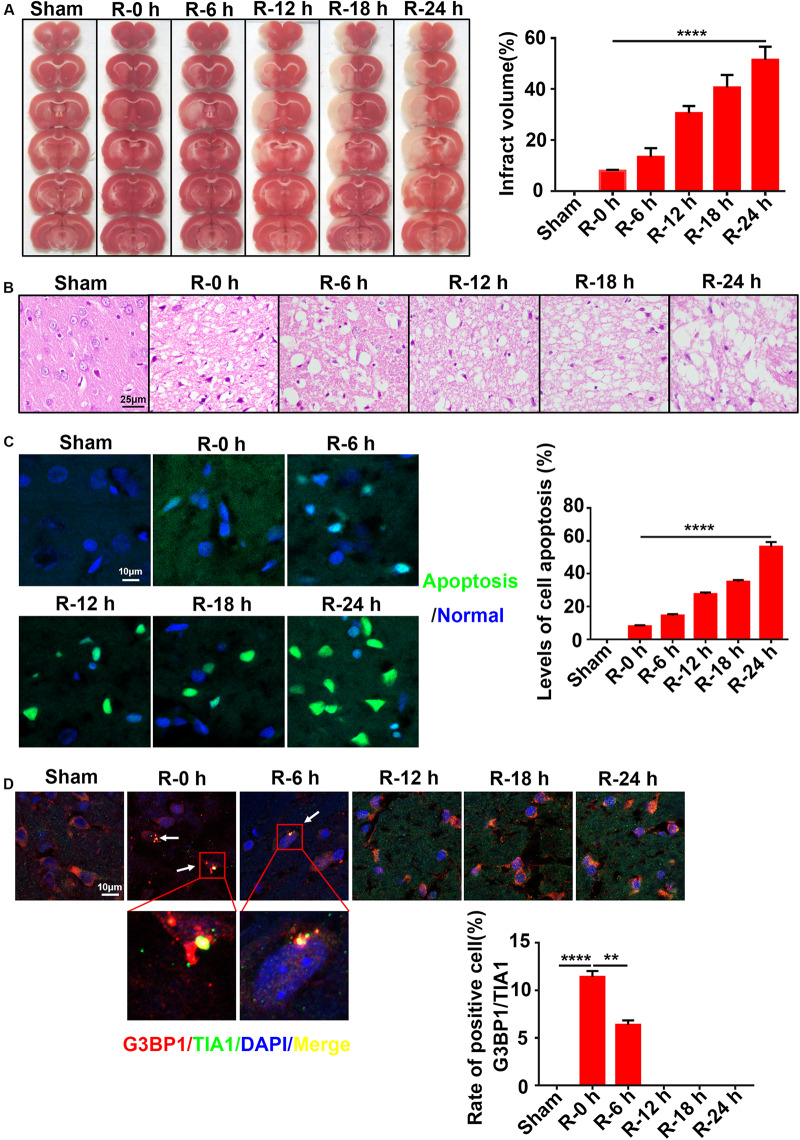
The inverse relation between SG formation and ischemic injury in ischemic cortex of MCAO rats. **(A)** Infarct volume was the greatest after 24 h of reperfusion in the MCAO model. TTC staining was used to measure infarct size (*n* = 72 rats, 12 rats/group). **(B)** The most severe brain injury appeared at 24 h post-reperfusion. **(C)** Apoptosis levels were elevated in the ischemic cortices of MCAO rats after 24 h of reperfusion. Green color represents apoptotic cells, and blue color represents DAPI staining for the nucleus. **(D)** SG formation was increased in the MCAO model after 0 and 6 h of reperfusion. SG labeled with TIA1 (green), G3BP1 (red), and DAPI (blue). Scale bars: 10 and 25 μm. SG, stress granules; MCAO, middle cerebral artery occlusion. **P value < 0.01; ****P value < 0.0001.

To explore the dynamic relation between SG formation and apoptosis level, rat brain tissues (*n* = 72 rats,12 rats/group) were obtained at different time points of reperfusion (0, 6, 12, 18, and 24 h) after 2 h MCAO surgery and assayed by TTC, Hematoxylin and Eosin (HE), TUNEL, and immunofluorescence staining. The results revealed that the apoptosis levels and infarct volume of ischemic cortices gradually increased from 0 to 24 h post-reperfusion (Figures 1A,C). The apoptosis level was significantly increased by more than fivefold (547.1%) in the R-24 h group (24 h post-reperfusion) (Figure 1A; R-0 h vs. R-24 h; *P* < 0.0001), and the infarct volume was increased by sixfold (601.2%) in the R-24 h group (Figure 1C; R-0 h vs. R-24 h; *P* < 0.0001) compared with the R-0 h group (0 h post-reperfusion). HE staining results showed that the level of necrosis in the cortex was proportional to the time points of post-reperfusion, which peaked at 24 h post-reperfusion (Figure 1B). However, SG formation was negatively correlated with the apoptosis level and ischemic injury in the cortex. According to the results of immunofluorescence staining, SG formation was observed in the ischemic cortical at 0 and 6 h post-reperfusion. Compared with the R-6 h group, SG formation significantly increased by 82.2% in the R-0 h group (Figure 1D; R-0 h vs. R-6 h; *P* < 0.01). These data suggested that SG formation occurred in the early stage of AIS injury (R-0 h) and disappeared in the later stage of the injury (R-24 h).

### Expression Levels of METTL3, m6A, and miR-335 in Ischemic Cortices of MCAO Rats at Different Time Points of Reperfusion

**FIGURE 2:**
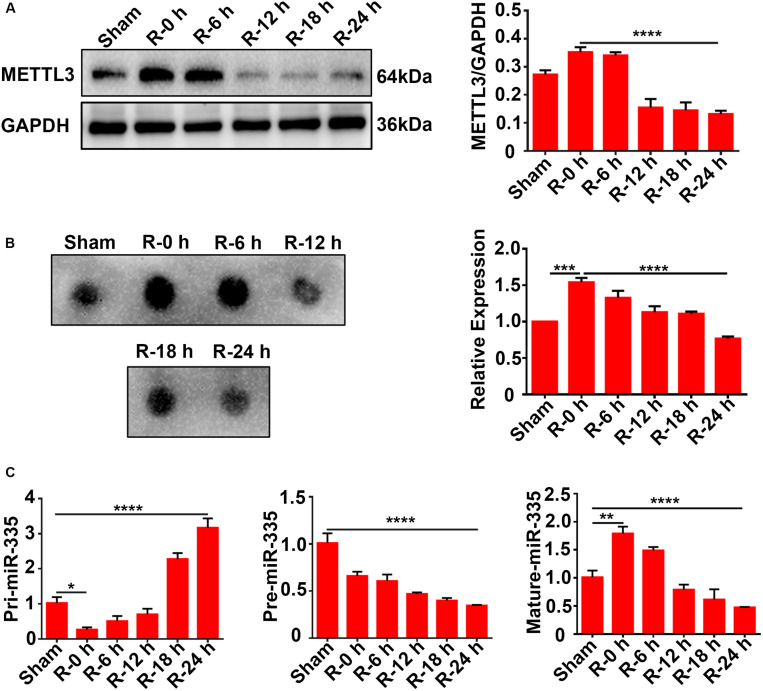
Expression levels of METTL3, m6A, and miR-335 in ischemic cortices of MCAO rats at different times of reperfusion (*n* = 36 rats, 6 rats/group). **(A)** Expression levels of METTL3 in the cortices of MCAO rats at different times of reperfusion. Rat cortices were obtained at different times of reperfusion (0, 6, 12, 18, and 24 h) after 2 h of MCAO surgery. **(B)** m6A levels of total RNA in cortices of MCAO rats at different times of reperfusion. **(C)** Pri-, Pre, and Mature-miR-335 expression levels in cortices of MCAO rats at different times of reperfusion. m6A, N6-methyladenosine. *P value < 0.05; **P value < 0.01; ***P value < 0.001; ****P value < 0.0001.

Rat cortices (*n* = 36 rats, six rats/group) were obtained to explore the expression levels of METTL3, m6A, and miR-335 at different time points of reperfusion (0, 6, 12, 18, and 24 h) after 2 h of MCAO surgery. Total protein and RNA were extracted from the ischemic cortex and subjected to western blot analysis, dot blot, and RT-qPCR assays. The results of the western blot analysis showed that METTL3 expression was transiently increased at R-0 h and R-6 h and then significantly decreased in the R-12 h, R-18 h, and R-24 h groups. METTL3 expression significantly decreased by 62.5% in R-24 h group (Figure 2A; R-0 h vs. R-24 h; *P* < 0.0001) compared with R-0 h group. A similar trend was observed for the m6A expression level of total RNA (Figure 2B; sham vs. R-0 h; *P* < 0.001; R-0 h vs. R-24 h; *P* < 0.0001). As presented in Figure 2C, the mature-miR-335 expression level was significantly increased by 83.4% in R-0 h group and decreased by 51.6% in R-24 h group compared with the sham group (Figure 2C; sham vs. R-0 h; *P* < 0.01; sham vs. R-24 h; *P* < 0.0001). The pri-miR-335 expression level was significantly decreased by 73.4% in the R-0 h group and increased more than twofold (217.7%) in the R-24 h group, compared with the sham group (Figure 2C; sham vs. R-0 h; *P* < 0.05; sham vs. R-24 h; *P* < 0.0001). In addition, pre-miR-335 expression level significantly decreased by 65.1% in the R-24 h group compared with the sham group (Figure 2C; sham vs. R-24 h; *P* < 0.0001). These data suggested that the expression levels of METTL3, m6A, and miR-335 increased in parallel during the early stage of AIS injury (R-0 h) and decreased in the later stage of AIS injury (R-24 h).

### The Inverse Relation Between SG Formation and Apoptosis Levels in PC12 Cells and Primary Cortical Neurons Under OGD/R Stimulation

**FIGURE 3:**
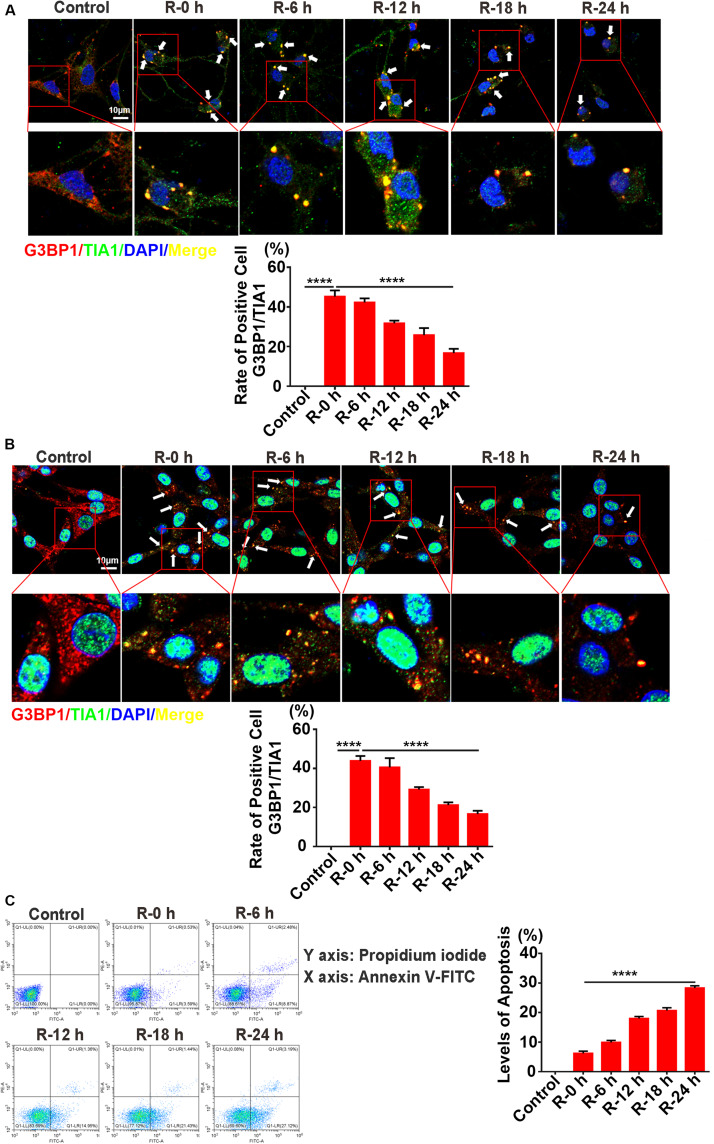
The inverse relation between SG formation and apoptosis levels in PC12 cells and primary cortical neurons under OGD/R stimulation. **(A)** SG formation in primary cortical neurons treated with different times of reperfusion (0, 6, 12, 18, and 24 h) after 6 h of OGD stimulation. SG labeled with TIA1 (green), G3BP1 (red), and DAPI (blue). **(B)** SG formation in PC12 cells treated with different times of reperfusion (0, 6, 12, 18, and 24 h) after 6 h of OGD stimulation. **(C)** Apoptosis levels in PC12 cells treated with different times of reperfusion (0, 6, 12, 18, and 24 h) after 6 h of OGD stimulation. Scale bars: 10 μm. ****P value < 0.0001.

To verify the results observed *in vivo*, we performed OGD/R (oxygen-glucose deprivation/recovery) stimulation in PC12 cells and primary cortical neurons as an *in vitro* model of AIS. Double-labeled immunofluorescence (TIA1 and G3BP1) and flow cytometry assays were used to observe SG formation and apoptosis levels at different time points of recovery (0, 6, 12, 18, and 24 h) after 6 h of OGD stimulation. According to the results of the immunofluorescence, SG formation was significantly increased more than twofold (166.9%) in R-0 h group in PC12 cells (Figure 3A; R-0 h vs. R-24 h; *P* < 0.0001) and increased almost twofold (184.1%) in the R-0 h group in primary cortical neurons (Figure 3B; R-0 h vs. R-24 h; *P* < 0.0001) compared with R-24 h group. In addition, flow cytometry analysis (annexin V/PI double staining) confirmed the inverse relation between SG formation and apoptosis levels *in vitro*. The apoptosis level was significantly reduced by 77.9% in the R-0 h group (Figure 3C; R-0 h vs. R-24 h; *P* < 0.001) compared with R-24 h group. These data suggested that SG formation occurred in the early stage of OGD/R stimulation (R-0 h) and decreased along with reperfusion period in PC12 cells and primary cortical neurons.

### Expression Levels of METTL3, m6A, and miR-335 in PC12 Cells Under OGD/R Stimulation

**FIGURE 4:**
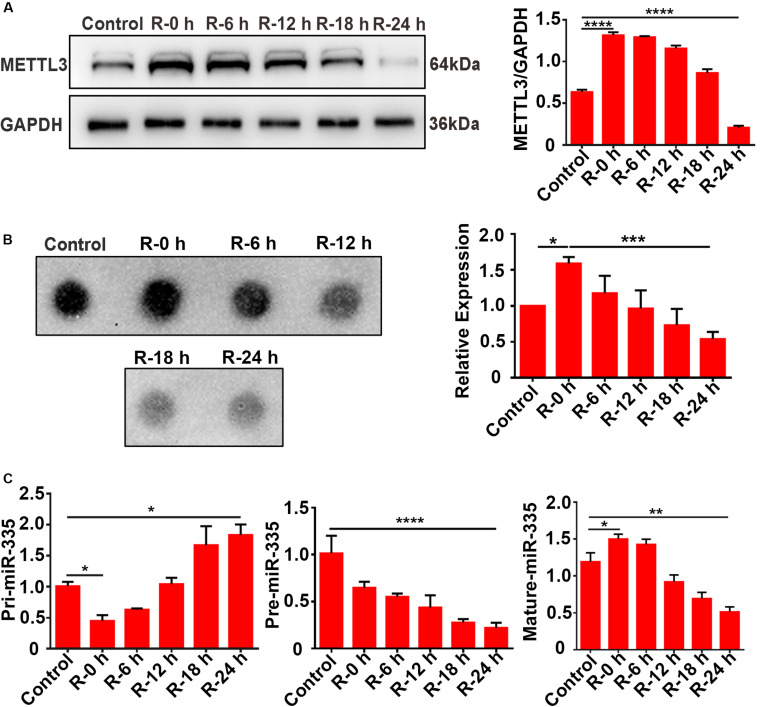
Expression levels of METTL3, m6A and miR-335 in PC12 cells at different times of reperfusion. **(A)** Expression levels of METTL3 in PC12 cells at different times of reperfusion. PC12 cells were treated with different times of reperfusion (0, 6, 12, 18, and 24 h) after 6 h of OGD stimulation. **(B)** m6A levels of total RNA in PC12 cells at different times of reperfusion. **(C)** Pri-, Pre-, and Mature-miR-335 expression levels in PC12 cells at different times of reperfusion. *P value < 0.05; **P value < 0.01; ***P value < 0.001; ****P value < 0.0001.

To verify the results observed *in vivo*, total protein and RNA were extracted from PC12 cells at different time points of recovery (0, 6, 12, 18, and 24 h) after 6 h of OGD stimulation and subjected to western blot analysis, dot blot, and RT-qPCR assays. The results of the western blot analysis showed that METTL3 expression significantly increased more than twofold (108.3%) in the R-0 h group compared with the control group, while it decreased by 66.9% in the R-24 h group (Figure 4A; control vs. R-0 h; *P* < 0.0001; control vs. R-24 h; *P* < 0.0001). In addition, a similar trend was observed for the m6A expression level of total RNA (Figure 4B; control vs. R-0 h; *P* < 0.05; R-0 h vs. R-24 h; *P* < 0.001). As presented in Figure 4C, the mature-miR-335 expression level was significantly increased by 29.7% in R-0 h group and decreased by 54.8% in R-24 h group (Figure 4C; control vs. R-0 h; *P* < 0.05; control vs. R-24 h; *P* < 0.01) compared with the control group. The pri-miR-335 expression level was significantly decreased by 56.2% in the R-0 h group and increased by 81.6% in the R-24 h group (Figure 4C; control vs. R-0 h; *P* < 0.05; control vs. R-24 h; *P* < 0.05), and the pre-miR-335 expression level was significantly decreased by 76.9% in the R-24 h group (Figure 4C; control vs. R-24 h; *P* < 0.0001) compared with control group. These *in vitro* results were correlated with the *in vivo* results shown in Figure 2, which suggested that the expression levels of METTL3, m6A, and miR-335 increased in parallel during the early stage of OGD/R injury (R-0 h) and decreased in the later stage of OGD/R injury (R-24 h).

### METTL3 Promotes the Relative m6A Level of pri-miR-335, Mature-miR-335 Expression, and SG Formation in PC12 Cells Under OGD Stimulation

To address the functional role of METTL3 in regulating miR-335 expression and SG formation of PC12 cells under OGD/R stimulation, CRISPR/Cas9 gene-editing system, m6A immunoprecipitation (MeRIP), RT-qPCR, and immunofluorescence assays were used. The results of RNA-binding protein immunoprecipitation (RIP) experiment showed that no significant direct connections between METTL3 and Erf1 mRNA and it was no significant changes between normal condition and 6 h of OGD stimulation (Supplementary Material S1).

#### METTL3 Promotes the Relative m6A Level of pri-miR-335 in PC12 Cells Under OGD Stimulation

**FIGURE 5:**
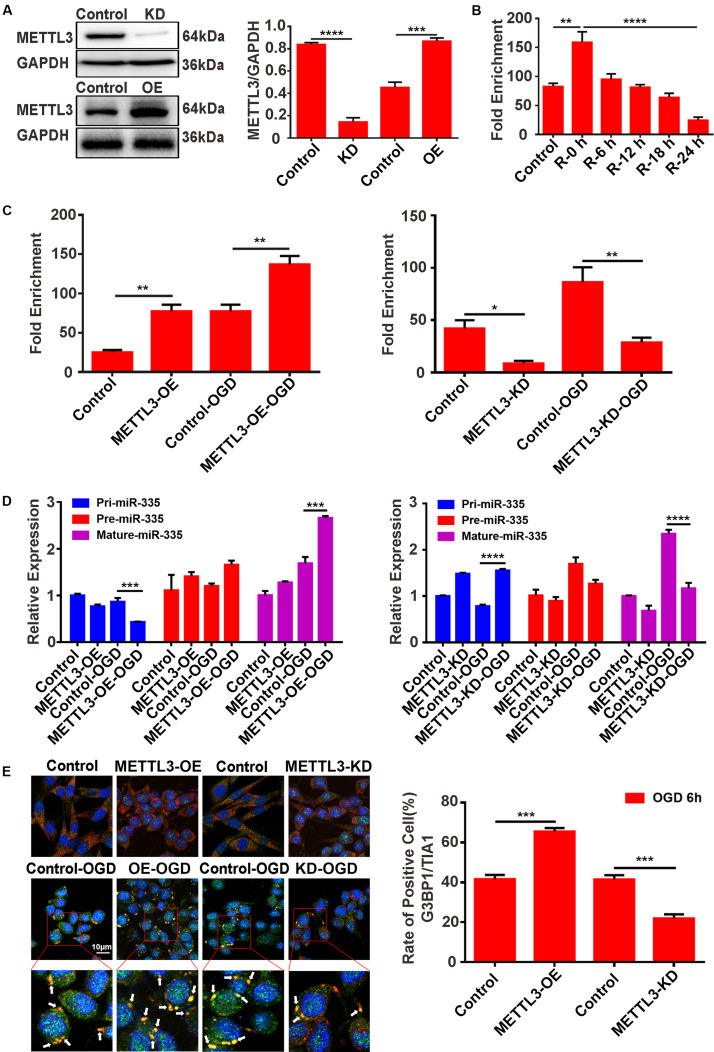
METTL3 promotes the m6A level of pri-miR-335, mature-miR-335, and SG formation in PC12 cells under OGD stimulation. **(A)** The efficiency of METTL3 knockdown or overexpression in PC12 cells. Western blot assays were used for validation. **(B)** m6A levels of total RNA in PC12 cells at different times of reperfusion. PC12 cells were treated for different times of reperfusion (0, 6, 12, 18, and 24 h) after 6 h of OGD stimulation. **(C)** The m6A level of pri-miR-335 in METTL3 knockdown or overexpression PC12 cells under OGD stimulation. **(D)** The expression level of pri-, pre-, and mature-miR-335 in METTL3 knockdown or overexpression PC12 cells under OGD stimulation. (control-OGD vs. METTL3-OE-OGD; *P* < 0.001; control-OGD vs. METTL3-KD-OGD; *P* < 0.0001). **(E)** SG formation in METTL3 knockdown or overexpression PC12 cells under OGD stimulation (control vs. METTL3-OE; *P* < 0.001; control vs. METTL3- KD; *P* < 0.001). *P value < 0.05; **P value < 0.01; ***P value < 0.001; ****P value < 0.0001.

METTL3 knockdown (METTL3-KD) and METTL3 overexpression (METTL3-OE) PC12 cells were generated using the CRISPR/Cas9 gene-editing system and transfection of the overexpression plasmid, respectively. Western blot assays validated the knockdown and overexpression efficiency of METTL3 (Figure 5A, control vs. KD; *P* < 0.0001; control vs. OE; *P* < 0.001). We first analyzed the relative m6A level of pri-miR-335 in PC12 cells under different reperfusion time points (0, 6, 12, 18, and 24 h) after 6 h of OGD stimulation. The results of the MeRIP showed that the relative m6A level of pri-miR-335 was the highest in the R-0 h group and the lowest in the R-24 h group (Figure 5B; control vs. R-0 h; *P* < 0.01; R-0 h vs. R-24 h; *P* < 0.0001). This trend was consistent with the change in METTL3 protein expression, relative m6A level of total RNA, and mature-miR-335 expression shown in Figures 4A–C. Then, the MeRIP assay was used to analyze the relative m6A level of pri-miR-335 in METTL3 KD/OE cells under 6 h of OGD stimulation. The relative m6A level of pri-miR-335 were significantly increased more than twofold (215.1%) in the METTL3-OE group and by 81.1% in the METTL3-OE-OGD group compared to the control and control-OGD groups, respectively (Figure 5C, control vs. METTL3-OE; *P* < 0.01; control-OGD vs. METTL3-OE-OGD; *P* < 0.01). The relative m6A level of pri-miR-335 was significantly decreased by 79.6% in the METTL3-KD and by 66.7% in the METTL3-KD-OGD groups (Figure 5C, control vs. METTL3-KD; *P* < 0.05; control-OGD vs. METTL3-KD-OGD; *P* < 0.01) compared to control and control-OGD groups, respectively.

#### METTL3 Promotes Mature-miR-335 Expression in PC12 Cells Under OGD Stimulation

Furthermore, RT-qPCR assays were used to detect the level of pri-, pre-, and mature-miR-335 expression. The results showed that the pri-miR-335 expression was significantly decreased by 49.2% in the METTL3-OE-OGD group and increased by 99.3% in the METTL3-KO-OGD group (Figure 5D, control-OGD vs. METTL3-OE-OGD; *P* < 0.001; control-OGD vs. METTL3-KO-OGD; *P* < 0.0001), compared with the control-OGD group. The mature-miR-335 expression was significantly increased by 59.5% in the METTL3-OE-OGD group and decreased by 50.1% in the METTL3-KO-OGD group (Figure 5D, control-OGD vs. METTL3-OE-OGD; *P* < 0.001; control-OGD vs. METTL3-KO-OGD; *P* < 0.0001) compared with respective control-OGD group.

#### METTL3 Promotes SG Formation in PC12 Cells Under OGD Stimulation

Double-labeled immunofluorescence (TIA1 and G3BP1) was used to detect SG formation in PC12 cells. The immunofluorescence results demonstrated that SG formation in the METTL3-OE group was 57.6% higher than that in the control group (Figure 5E, control vs. METTL3-OE; *P* < 0.001), while SG formation in the METTL3-KO group was 46.5% lower than that of control group after 6 h of OGD stimulation (Figure 5E, control vs. METTL3-KO; *P* < 0.001).

These data suggested that METTL3 played a vital role in regulating the relative m6A level of pri-miR-335, mature-miR-335 expression, and SG formation.

### MiR-335 Specifically Decreases Erf1 Expression, Promotes SG Formation, and Prevents Cell Apoptosis in PC12 Cells Under OGD Stimulation

**FIGURE 6:**
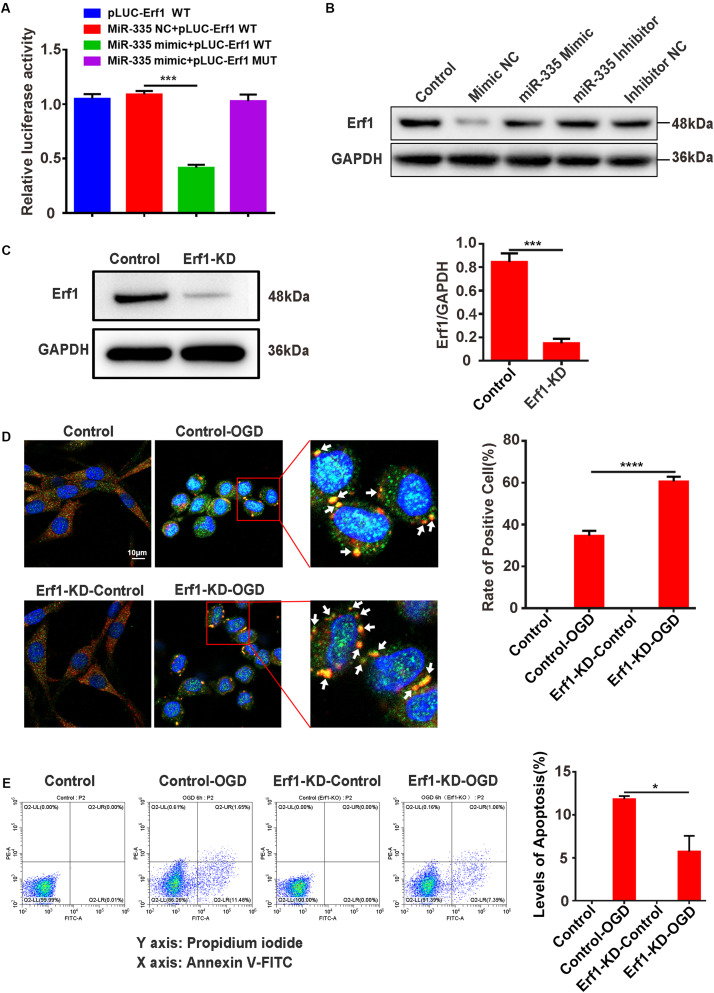
MiR-335 specifically decreases Erf1 expression, promotes SG formation, and prevents cell apoptosis levels in PC12 cells under OGD stimulation. **(A)** MiR-335 directly targeted the 3′-UTR of Erf1 mRNA in PC12 cells. The results of the dual-luciferase reporter system assay are shown in figure. **(B)** Erf1 protein expression was decreased in PC12 cells transfected with miR-335 mimic. **(C)** Efficiency of Erf1 knockdown in PC12 cells. **(D)** SG formation was elevated in Erf1-knockdown PC12 cells under OGD stimulation. Cells were stimulated for 6 h under OGD conditions. **(E)** The apoptosis level was reduced in Erf1-knock PC12 cells under OGD stimulation. Annexin V-FITC/PI double staining assay was used for apoptosis levels of cells detection. X axis: PI; Y axis: Annexin V. control-OGD vs. Erf1-KD-OGD; *P* < 0.05. *P value < 0.05; ***P value < 0.001; ****P value < 0.0001.

The predicted results of the bioinformatics website (Target Scan) showed that miR-335 had two predicted binding sites in the 3′-UTR of Erf1 mRNA; the profile of pLUC-Erf1-WT and pLUC-Erf1-MUT are presented in Supplementary Material S3. The results of the dual-luciferase reporter system assay showed that the relative luciferase activity was significantly decreased by 61.7% in pLUC-Erf1 + miR-335 mimic group [Figure 6A; miR-335 mimic NC + pLUC-Erf1 WT vs. miR-335 mimic + pLUC-Erf1 WT; *P* < 0.001; negative control (NC)] compared with the miR-335 mimic NC + pLUC-Erf1 WT group. This result indicated that the 3′-UTR of Erf1 mRNA was the target of miR-335 in PC12 cells. Western blotting analysis showed that the protein expression of Erf1 in PC12 cells was significantly reduced by 56.8% by transfecting miR-335 mimic as against miR-335 mimic NC (Figure 6B). This result validated the results of the dual-luciferase reporter system assay. Erf1-knockdown PC12 cells were generated using the CRISPR/Cas9 gene-editing system, and western blot assays validated the knockdown efficiency of Erf1 in PC12 cells (Figure 6C). Furthermore, the immunofluorescence results demonstrated that SG formation in the Erf1-KD-OGD group was 77.2% higher than that of the control-OGD group (Figure 6D, control-OGD vs. Erf1-KD-OGD; *P* < 0.0001), which demonstrated that Erf1 played an essential role in SG formation under OGD conditions. In addition, apoptosis levels of cells were determined by an annexin V-FITC/PI double staining assay. As shown in Figure 6E, the apoptosis level of cells was significantly reduced by 50.7% in the Erf1-KD-OGD group (Figure 6E, control-OGD vs. Erf1-KD-OGD; *P* < 0.05) compared with the control-OGD group. These results indicated that miR-335 decreased Erf1 protein expression, promoted SG formation, and prevented cell apoptosis in PC12 cells under OGD stimulation.

## Discussion

Here, we identified an important role of METTL3-mediated m6A modification in promoting SG formation by upregulating the expression of mature miR-335 which targets the eukaryotic translation termination factor (Erf1). METTL3-mediated SG formation is closely associated with improving various pathological conditions and represents a promising strategy for the treatment of AIS. The identification of METTL3, as a novel positive regulator of SG formation, may significantly increase our understanding of the pathogenesis of AIS. The METTL3/miR-335/Erf1 axis was found to contribute to SG formation, which may enable the development of therapeutic strategies for the prevention and treatment of AIS injury in the early stage.

SGs are crucial for facilitating cell survival under stress conditions (Hofmann et al., 2012; Kim et al., 2012; Chang and Huang, 2014). To our knowledge, we identified for the first time, SG formation in the ischemic cortical of MCAO rats and in the oxygen-glucose deprivation of PC12 cells using double-labeled immunofluorescence (TIA1 and G3BP1). In our previous publication we showed that SG formation may reduce ischemic cortical injury in the early stage of AIS (Si et al., 2019). Therefore, our *in vitro* and *in vivo* experimental results suggested that METTL3-mediated SG formation might reduce AIS injury in the early stage of the disease.

An important finding of our study is that METTL3-mediated m6A modification promoted SG formation at an early stage of AIS. A study showed that m6A expression is higher in the brain than in other tissues (Meyer and Jaffrey, 2014), which suggested that the tissue specificity of m6A expression is in the brain. The m6A methyltransferase, known as “Writers,” is an important catalytic enzyme (Meyer and Jaffrey, 2017; Gu et al., 2020; Yu et al., 2020). m6A methylation has a wide range of effects on the nervous system, learning memory, brain development and synaptic growth (Engel and Chen, 2018; Ji et al., 2018; Shi and He, 2018; Widagdo and Anggono, 2018). However, m6A methylation is a relatively new field and its functions in AIS remains unknown. Our results are consistent with previous findings that m6A methylation facilitates mRNA triaging to stress granules (Anders et al., 2018). In contrast to the static m6A modification (Cao et al., 2016; Huisman et al., 2017; Tong et al., 2018), we found that the METTL3-mediated SG formation is dynamic. Identification of the target of METTL3 is an important next step to explore the regulation mechanisms of SG formation.

The mechanism by which METTL3-mediated SG formation exerts its positive effects on AIS is through miR-335/Erf1 signaling. Studies have shown that METTL3 influences the biogenesis process of miRNAs by regulating the methylation level of the primary transcription of miRNAs, and finally regulates the expression level of mature microRNAs (Alarcon et al., 2015; Erson-Bensan and Begik, 2017; Han et al., 2019). However, few studies have focused on the regulation of METTL3 to the methylation level of pri-mir-335 in AIS. In this study, we found that METTL3 regulated the m6A level of pri-miR-335 and increased the expression of mature-miR-335 in AIS. The results further confirmed the critical role of METTL3 in the biogenesis processing of miRNAs. Multiple lines of evidence presented here indicate that the catalytic activity of METTL3 mediates the SG formation.

In the present study, we found that miR-335 might specifically decrease the expression of Erf1 to increase SG formation. As a eukaryotic translation termination factor, Erf1 protein expression reduction represents an increasing level of translation arrest, which promotes SG formation (Cheng et al., 2009; Grousl et al., 2013; Blanchet et al., 2015). The results of the present study indicated that the METTL3/miR-335/Erf1 axis contributed to SG formation in the early stage of AIS, which may enable the development of new therapeutic strategies for the prevention and treatment of the early stage of the disease.

The results of the present study have several important clinical implications. First, stroke is one of the main causes of morbidity and long-term disability worldwide. There is an urgent demand for sensitive and specific biomarkers. Our previous studies showed that miR-335 plays an essential role in acute stroke in animal and cell models and is sensitive to AIS injury (Si et al., 2019). It has been reported that the specific expression of miR-335 is changes significantly in the serum of patients with acute stroke (Liu et al., 2015; Zhao et al., 2016). Thus, the miR-335 may be a potential biomarker for the early pathogenesis of stroke. Second, our results showed that METTL3-mediated SG formation is a promising strategy for the treatment of AIS. Currently, there are few therapeutic targets to protect against the progression of AIS. SGs are of interest as therapeutic targets to AIS since they play an important role in the cell viability of neurons under stress response (Barbee et al., 2006).

## Conclusion

Our study suggested that METTL3-mediated m6A modification plays an important role in promoting SG formation and decrease AIS injury in the early stage of the condition. Additionally, our results further indicated that METTL3 interacting with the miR-335/Erf1 signaling pathway promotes SG formation. The METTL3/miR-335/Erf1 axis may represent a targeted and mechanism-based therapeutic strategy against brain damage following AIS.

## Data Availability Statement

The datasets generated for this study are available on request to the corresponding author.

## Ethics Statement

The animal study was reviewed and approved by the Ethical Committee of the Guangzhou University of Chinese Medicine.

## Author Contributions

WS performed the cell and animal experiments and wrote the manuscript. SY performed the western blotting assays. YiL performed the immunofluorescence assay. WS and ZL performed the RNA immunoprecipitation and CRISPR/Cas9 gene-editing assays. YaL performed the flow cytometry analysis. WK provided the reagents and materials. MZ and DC designed the experiments and gave experimental technical guidance. All authors have read and approved the final manuscript.

## Conflict of Interest

The authors declare that the research was conducted in the absence of any commercial or financial relationships that could be construed as a potential conflict of interest.

## Footnotes

1 http://crispr.mit.edu
