# Supplementary material for: Methyltransferase 3 Mediated miRNA m6A Methylation Promotes Stress Granule Formation in the Early Stage of Acute Ischemic Stroke
Source: Front Mol Neurosci. 2020 Jun 5;13:103. doi: 10.3389/fnmol.2020.00103 (PMC7289951; doi:10.3389/fnmol.2020.00103)
Supplement: Supplementary file 1 [file Data_Sheet_1.PDF]

Supplementary Material S1

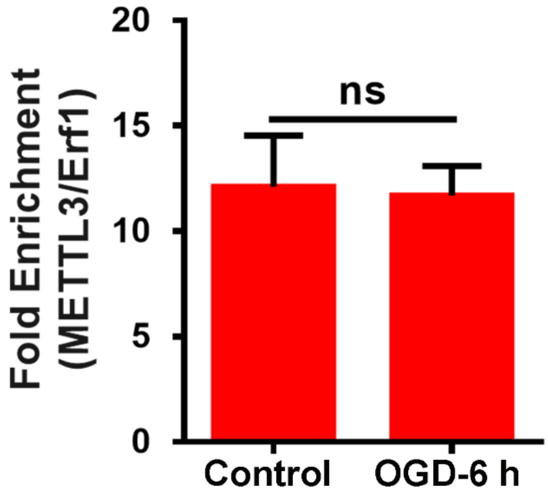

Figure S1 RNA-binding protein immunoprecipitation (RIP) experiment was used to assess the binding interaction between METTL3 and Erf1 mRNA under normal and OGD conditions (6 h of OGD stimulation). The results showed that the binding interaction was low (control group: 12.11 ± 2.43; OGD-6 h group: 11.70 ± 1.40) and it was no significant changes between normal condition and OGD stimulation (ns: no significant difference). As such, no significant direct connections between METTL3 and Erf1 mRNA. SPSS 20.0 software (IBM Corp., NY, USA) was used for statistical analyses. The normality of values was tested with the Shapiro–Wilk normality test. The figure was produced using GraphPad Prism (Version 6.0; GraphPad Software, Inc., La Jolla, CA, USA). A two-tailed unpaired t-test or non-parametric Mann–Whitney U-test was used to analyze the difference between the two groups. A p-value of < 0.05 was considered statistically significant. Data are presented as the mean ± SEM. Three independent experiments were performed with similar results.
