# Supplementary material for: Methyltransferase 3 Mediated miRNA m6A Methylation Promotes Stress Granule Formation in the Early Stage of Acute Ischemic Stroke
Source: Front Mol Neurosci. 2020 Jun 5;13:103. doi: 10.3389/fnmol.2020.00103 (PMC7289951; doi:10.3389/fnmol.2020.00103)
Supplement: Supplementary file 2 [file Data_Sheet_2.PDF]

Supplementary Material S2

	Name	Forward sequence (5′-3′)	Reverse sequence (5′-3′)
1	METTL3-sgRNA	accgcttagggccactagaggta	aaactacctctagtggccctaag
2	METTL3-sg-PCR	taaggaatccagaagcagcact	caccatgaacaaaagcaaatgt
3	Erf1-sgRNA	accgctcattaagagcttggagg	aaaccctccaagctcttaatgag
4	Erf1-sg-PCR	actctgtggtcttggtgtctcc	gctactacccagaggtccagaa

Table S2 Primers used for METTL3 and Erf1 Knockdown in PC12 cells. METTL3-sgRNA and Erf1-sgRNA were the complementary oligonucleotides of the target sequences of METTL3 and Erf1 with BsaI restriction sites (small guide RNAs, sgRNAs) and inserted into the pGL3-U6-gRNA plasmid. METTL3-sg-PCR and Erf1-sg-PCR were used for the amplification of target sequences and then sent for DNA sequencing analysis.

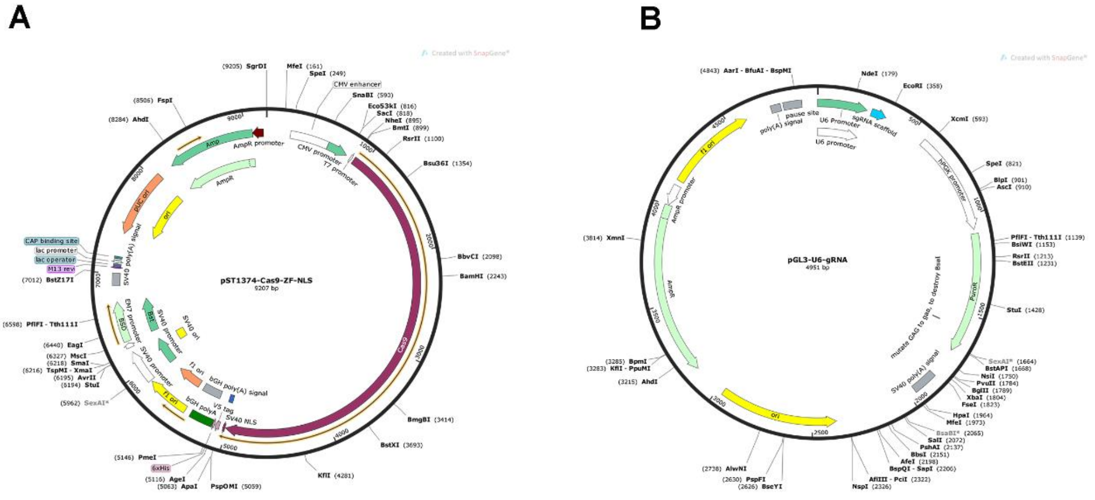

Figure S2 The plasmid maps used for METTL3 and Erf1 Knockdown. (A) The plasmid maps of pST1374-Cas9-ZF-NLS. (B) The plasmid maps of pGL3-U6-gRNA.
