# Supplementary material for: Methyltransferase 3 Mediated miRNA m6A Methylation Promotes Stress Granule Formation in the Early Stage of Acute Ischemic Stroke
Source: Front Mol Neurosci. 2020 Jun 5;13:103. doi: 10.3389/fnmol.2020.00103 (PMC7289951; doi:10.3389/fnmol.2020.00103)
Supplement: Supplementary file 3 [file Data_Sheet_3.PDF]

Supplementary Material S3

A

Mutation sites 1:

3' UGUAAAAAGCAAUAACGAGAACU 5' rno-miR-335

||||||||

5'...AUGGUAAAAUUGGUUGCUCUUGU...3' Erf1-WT (3'UTR)

5'...AUGGUAAAAUUGGUUCGAGAACU...3' Erf1-Mut (3'UTR)

Mutation sites 2:

3' UGUAAAAAGCAAUAACGAGAACU 5' rno-miR-335

||||||||

5'...CAACGCUUGUGCCCGGCUCUUGG...3' Erf1-WT (3'UTR)

5'...CAACGCUUGUGCCCGCGAGAACG...3' Erf1-Mut (3'UTR)

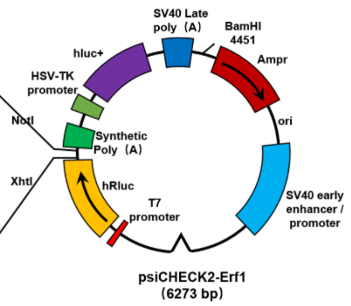

B

3′-UTR sequence of psiCHECK2-ETF1-WT

CAGCAGCCTACAACCAAGTCCCTAAATGCCACTTTGGACTAATTTAAAAGAATCCCAGTTT
TTACTTTTACTTGATGGTAAAATTGGTTGCTCTTGTATTTTATGAAAAAAATGATTTTTTTAA
CTTCATACATAGAAGCAAAATACTTTAACTGCTGTAAACCTTCAGAAGTCAGTAGACGTGA
GATCATACTGGTTGGTTTCTCCCTCTGATCGGAGAGTAACTAAATTGCTGCGTTCGCTGC
CTCCGCAGTGACCCATTTACATGGCGTTCTCGGCTTAGACTGCACAAGAAGAAATATATG
GGTGAAATGTTGGAACCGTTTCTCTCTTGGTCTCTGTTTAATGTTGAAAAGGGTGAGCTA
TAGGCAATTTCAGTTTCACTCCCTCACACCCCCACTCCCAGACTGTCCGTTTCAAGGGTG
AGGTTGTGTTGTAAACTCAGACTGAGTGCGGAAACACGGGGCCAGTGCACTGTTGGCTT
CATCAGTGTGGGGGCAACGCTTGTGCCCGGCTCTTGGGAGACGGTGCTCTACTGAGGC
TCCCTATAACCTCAGCCTACTAGAAACCAGTCTGGGATGGATATATGGGGCTTCT

C

3′-UTR sequence of psiCHECK2-Erf1-Mut

CAGCAGCCTACAACCAAGTCCCTAAATGCCACTTTGGACTAATTTAAAAGAATCCCAGTTT
TTACTTTTACTTGATGGTAAAATTGGTTCGAGAACTATTTTATGAAAAAAATGATTTTTTTAA
CTTCATACATAGAAGCAAAATACTTTAACTGCTGTAAACCTTCAGAAGTCAGTAGACGTGA
GATCATACTGGTTGGTTTCTCCCTCTGATCGGAGAGTAACTAAATTGCTGCGTTCGCTGC
CTCCGCAGTGACCCATTTACATGGCGTTCTCGGCTTAGACTGCACAAGAAGAAATATATG
GGTGAAATGTTGGAACCGTTTCTCTCTTGGTCTCTGTTTAATGTTGAAAAGGGTGAGCTAA
TAGGCAATTTCAGTTTCACTCCCTCACACCCCCACTCCCAGACTGTCCGTTTCAAGGGTG
AGGTTGTGTTGTAAACTCAGACTGAGTGCGGAAACACGGGGCCAGTGCACTGTTGGCTT
CATCAGTGTGGGGGCAACGCTTGTGCCCGCGAGAACGGAGACGGTGCTCTACTGAGGC
TCCCTATAACCTCAGCCTACTAGAAACCAGTCTGGGATGGATATATGGGGCTTCT

Supplementary Figure S3. MiR-335 directly targeted the 3′-UTR of Erf1 mRNA in PC12 cells. (A) Prediction of major interference sites between miR-335 and Erf1 mRNA 3′-UTR by using Targetscan. The plasmid map of the psiCHECK2-Erf1, which included miR-335 binding sites, was shown in the right panel. (B) The 3′-UTR sequence of the psiCHECK2-Erf1-WT plasmid. Underlined sequences represented the binding sites between miR-335 and Erf1 mRNA 3′-UTR. (C) The 3′-UTR sequence of the psiCHECK2-Erf1-Mut plasmid. The underlined sequence was required to mutate. The mutated binding sites between miR-335 and Erf1 mRNA 3′-UTR were labelled by the red colour.
